# Bacterial bioluminescence onset and quenching: a dynamical model for a *quorum sensing*-mediated property

**DOI:** 10.1098/rsos.171586

**Published:** 2017-12-13

**Authors:** Domenico Delle Side, Vincenzo Nassisi, Cecilia Pennetta, Pietro Alifano, Marco Di Salvo, Adelfia Talà, Aleksei Chechkin, Flavio Seno, Antonio Trovato

**Affiliations:** 1 Dipartimento di Matematica e Fisica ‘Ennio De Giorgi’, Università del Salento, Lecce, Italy; 2 Dipartimento di Scienze e Tecnologie Biologiche ed Ambientali, Università del Salento, Lecce, Italy; 3 Istituto Nazionale di Fisica Nucleare, Sezione di Lecce, Lecce, Italy; 4 Akhiezer Institute for Theoretical Physics, Kharkov Institute of Physics and Technology, Kharkov 61108, Ukraine; 5 Institute of Physics and Astronomy, University of Potsdam, 14476 Potsdam, Germany; 6 Dipartimento di Fisica e Astronomia ‘Galileo Galilei’, Università di Padova, Padova, Italy; 7 Istituto Nazionale di Fisica Nucleare, Sezione di Padova, Padova, Italy

**Keywords:** quorum sensing, bioluminescence, biophysical model, Harveyi clade, oxygen quenching, Gompertz growth function

## Abstract

We present an effective dynamical model for the onset of bacterial bioluminescence, one of the most studied quorum sensing-mediated traits. Our model is built upon simple equations that describe the growth of the bacterial colony, the production and accumulation of autoinducer signal molecules, their sensing within bacterial cells, and the ensuing quorum activation mechanism that triggers bioluminescent emission. The model is directly tested to quantitatively reproduce the experimental distributions of photon emission times, previously measured for bacterial colonies of *Vibrio jasicida*, a luminescent bacterium belonging to the Harveyi clade, growing in a highly drying environment. A distinctive and novel feature of the proposed model is bioluminescence ‘quenching’ after a given time elapsed from activation. Using an advanced fitting procedure based on the simulated annealing algorithm, we are able to infer from the experimental observations the biochemical parameters used in the model. Such parameters are in good agreement with the literature data. As a further result, we find that, at least in our experimental conditions, light emission in bioluminescent bacteria appears to originate from a subtle balance between colony growth and quorum activation due to autoinducers diffusion, with the two phenomena occurring on the same time scale. This finding is consistent with a negative feedback mechanism previously reported for *Vibrio harveyi*.

## 1. Introduction

Many living organisms are able to transform chemical energy into visible light, an ability known as bioluminescence [1]. Light emission is due to a reaction involving molecular oxygen, occurring on a substrate (luciferin, in most cases) and catalysed by an enzyme (luciferase). Substrate and enzyme properties change significantly across different bioluminescent systems, the sole common features being light emission and the requirement for molecular oxygen. Bioluminescent bacteria are the most abundant and widely distributed light-emitting organisms [2,3]. In cases when bacteria grow as symbionts with fishes or squids, the function of light emission relates to the use of photogenic organs by the host, whereas bacteria receive nutrients. In these organisms, the light-emitting reaction involves a luciferase-catalysed oxidation of reduced flavin mononucleotide, with the concomitant oxidation of a long-chain aliphatic aldehyde. This leads to the emission of blue–green light from an electronically excited species.

The study of the onset of bioluminescence in bacterial colonies has led to the finding of the fascinating mechanism now generally called ‘quorum sensing’ (QS) [4]. QS is the mechanism by which bacteria are able to ‘sense’ their environment, by activating complex collective actions that result beneficial to bacterial cells only when carried out by a group. Besides bioluminescence, these actions involve the expression of genes that control biofilm development, virulence and several other traits [5]. There is a large knowledge about the biochemistry of QS [6]. It is widely known that bacteria realize this mechanism through the synthesis, secretion and detection of some chemical signalling molecules known as *autoinducers* (AIs). In particular, a key role is played by the AI concentration profile that is generated across the colony. QS-responsive bacteria are able to detect the attainment of a ‘quorum’ threshold concentration of AIs through their binding to dedicated receptors. This triggers an intracellular signalling cascade that results in a phenotypic switch. In this way, all cells in the colony can become ‘quorum-active’ and start new collective behaviours in a coordinate manner.

The evolutionary origin of QS is still being hotly debated. AI concentration was initially considered as a direct proxy for cell density. In fact, this concept is at the origin of the expression ‘quorum sensing’ [7]. However, it is now clear [5,8–16] that AI concentration could be affected by several environmental factors other than cell density, such as AI diffusion, advection, the spatial arrangement of bacteria and the colony extension from an adsorbing boundary [17]. Furthermore, it has been suggested that AIs could work also as transducers of host cues [18].

Lately, the QS mechanism attracted significant interest as a possible target for the development of drugs that interfere with AIs’ signalling in order to prevent biofilm development or the expression of virulence factors. In particular, it has been considered as a target for next-generation drugs [19,20] able to overcome the problems arising from the rapid increase of antibiotic-resistant bacterial diseases which have been observed over the last decades [21].

Despite the important scientific achievements obtained in the field of QS biochemistry, we still know very little about the generic features of the collective QS behaviour, and especially of its interplay with the growth of a bacterial colony. For example, it is practically impossible to answer the simple question ‘when will a growing bacterial colony reach QS?’. Several groups proposed interesting dynamical models to describe QS [22–27]. All such approaches, however, are based on a detailed description of the biochemical processes underlying QS, thus requiring a very large number of parameters, most of which are extremely difficult to know with a reasonable precision. Furthermore, some of the models available so far were developed when the biochemical details about QS had not been fully assessed yet.

In this work, we present a simplified effective dynamical model for the onset of bacterial bioluminescence. Our main aim is to disentangle the roles of colony growth and of quorum activation in shaping the bioluminescent signal. The model is directly tested to quantitatively reproduce the experimental distributions of emission times for photons emitted by growing bacterial colonies of *Vibrio jasicida*, a member of the Harveyi clade [28,29]. We use data that were previously measured for bacterial colonies growing in a highly drying environment [30]. This minimizes swarming and allows us to model bacteria as fixed on the growing substrate. This is a crucial simplification for our model that is missing or is unjustified in other approaches.

The same experimental data were observed to follow the extreme value Gumbel statistics [30]. However, the connection between this kind of statistical distribution and bioluminescence activation was hypothetical, and the Gumbel distribution parameters used to fit the data had no clear interpretation. The simplified model that we propose here is able to fit experimental bioluminescence data with a slightly better quality than the Gumbel statistics. Most importantly, all parameters used in the model have a definite biochemical meaning and the values we estimate for them are consistent with what is known in the literature. Moreover, a good fitting of experimental data crucially requires that all cells cease light emission after a typical ‘quenching time’ (approx. 30 s) elapsed since their own activation. Intriguingly, this novel feature might be part of an oxygen quenching mechanism that establishes a tight metabolic control over the amount of reactive oxygen species in the bacterial ‘milieu’ [31,32]. Finally, our model allows to show that the overall bioluminescent signal can be split into two roughly equally weighted contributions, one related to the growth of the colony and the other to the increase in the number of bioluminescent cells due to quorum activation. This rationalizes the highly nonlinear relationship between the total number of emitted photons and the number of bacterial cells previously observed during growth [33].

## 2. Experimental set-up

### 2.1. Vibrio jasicida

The collection of the bioluminescence data was performed against growing cultures of *V. jasicida*, a member of the Harveyi clade [28,29]. The core of the Harveyi clade presently consists of *Vibrio harveyi* and its closely related species *Vibrio campbellii*, *Vibrio owensii*, *V. jasicida* and *Vibrio rotiferianus* [34]. Their members are commonly used as models to study bacterial luminescence [35,36], QS [37], biofilm formation [38] and multi-chromosomal genome organization [39,40]. These bacteria play important roles in marine ecosystems because they establish mutualistic, commensalistic or parasitic symbiosis with a wide range of marine invertebrates and vertebrates [18,41–46]. In particular, *V. jasicida* has been isolated from packhorse lobster, abalone, Atlantic salmon [29], Polychaeta [46], and some Hydrozoa and Bryozoa species [28].

### 2.2. Measure of bioluminescence signal

The strain samples of *V. jasicida* were cultured on nutrient broth (Difco) containing 3% NaCl at 20^°^C to an optical density of 1.0 at 550 nm. A volume of 10 μl of the suspension was spotted on the centre of 3% NaCl nutrient agar plates and incubated at (30±1)^°^C inside a climate chamber under nearly constant temperature and humidity conditions. Absolute darkness was operated inside the chamber. The experimental set-up contained a photomultiplier tube (PMT) Hamamatsu 1P28 able to record the low light emitted by samples. Its gain factor was 9×10^5^, while the nominal PMT spectral sensibility ranged from 185 to 650 nm. Its active window, which we used to pick up the light emitted from samples, has a height of 24 mm and a width of 8 mm; moreover, the spot was positioned directly against the window at a distance of 35 mm. The photomultiplier signals were collected by a workstation interfaced to a personal computer used both as storage and for timing the measurements each 5 or 10 min. A channel of the workstation was used to record the temperature. It is worth noting that we used Petri dishes without cover in order to avoid any filtering effect from the composing plastic material.

## 3. A simplified model for bioluminescence onset and quenching

### 3.1. Model summary

In our model, we assume we are dealing with a bacterial strain able to synthesize and respond to a single AI. In a nutshell, we take into account (i) the growth of the bacterial colony; (ii) the production and degradation of AI molecules; (iii) the sensing of AI concentration above the quorum threshold that activates the bacteria and induces light emission; (iv) luminescence turning off after an elapsed ‘quenching’ time assumed to be the same for all cells; and (v) the production of photons that depends on the copy number of luciferase enzymes in bioluminescent bacteria and on the luciferase turnover number in the photon emission reaction.

### 3.2. Model description

Our first ingredient is a reliable and simple way to estimate the increase with time of the number of bacteria, *n*(*t*). This could be accomplished by choosing a suitable sigmoidal function that well approximates the bacterial growth curve. The *Gompertz function* is one of the most popular growth laws [47]. This function, in particular, is used to model the decimal logarithm of *n*(*t*)/*n*(0)

3.1 $$\log_{10}\left(\frac{n(t)}{n(0)}\right)=K\;\exp\;\left[-\exp\;\left(-\frac{\mu \cdot \exp(1)}{K}(t-\lambda )+1\right)\right].$$

Four parameters enter the growth function: the initial number of bacterial cells *n*(0), the carrying capacity *K*, the lag time λ and the maximum specific growth rate *μ*.

AI diffusion follows Fick’s second law. However, we do not consider any dependence on the spatial coordinates, as is expected for the case of a spatially homogeneous colony in the presence of reflecting boundary conditions [14]. Furthermore, a drying environment is known to discourage bacteria movements on agar plates [48,49], so that advection can be neglected as well. As a consequence, we assume that the time derivative of AI concentration, d*c*(*t*)/d*t*, depends only on the number of bacteria, considered as point-like sources, and on *c*(*t*) itself, due to AI degradation [50], as given by

3.2 $$\frac{dc(t)}{dt}=\frac{\alpha }{V}n(t)-\beta c(t).$$

The two relevant parameters are AI production rate *α* and AI degradation rate *β*. The density of sources in the system is given by *n*(*t*)/*V* , with *V* the volume of the drop deposited on the agar dish where AI diffusion is taking place. In describing bacteria as point-like sources, the transport time of AI through the bacterial membrane (approx. 20 s [51]) can be safely neglected, compared to the time interval between consecutive measurements of photon emission used in the experiments, 300 and 600 s [30].

Furthermore, in order to estimate the fraction *f*(*t*) of quorum-active bacteria at time *t*, we use the *Hill function* [52] of AI concentration

3.3 $$f(t)=\frac{1}{1+{\left[c^{*}/c(t)\right]}^{\delta }}.$$

The Hill function is generally used to model the equilibrium thermodynamic properties of the binding of ligand molecules to a receptor that produces a functional effect. The two relevant parameters in the Hill function are the ‘quorum’ threshold concentration c* (the dissociation constant of the AI–receptor complex) and the cooperativity index *δ* (*δ*>1 denotes a cooperative binding).

The number of active bacteria *q*(*t*) at time *t* is then given by

3.4 q(t)=f(t)⋅n(t).

**Figure 1.**
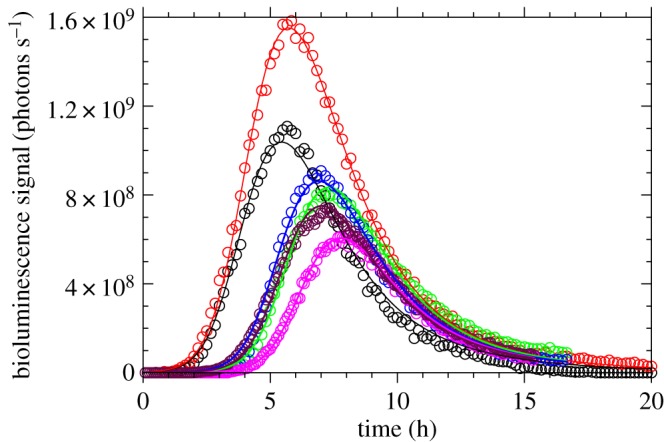
Experimental observations of the radiant flux emitted by bacteria (circles) as obtained in [30] and the corresponding fits (solid line) obtained by numerically solving equations (3.10)–(3.14). Different colours represent the different experimental curves measured in [30]. The raw data shown here are presented in fig. 3 of Delle Side *et al*. [30] as normalized distributions of *z*-scores with zero mean and unit variance.

Active bacteria start to express the genes controlling bioluminescence. This gives rise to a series of biochemical reactions in which part of the chemical energy involved is released as photons in the blue–green range. In the experiments considered here, the light emission by the colony increases and subsequently decays in course of time [30], as can be seen in figure 1. As the number of active bacteria *q*(*t*) is never decreasing, their bioluminescent emission cannot be constant and has to decay in course of time. We hypothesize that this decay can be described by a generic ‘memory function’ *m*(*t*,*t*′) that quantifies how much bioluminescent emission at time *t* is reduced for bacteria that were activated at a previous time *t*′≤*t*, with respect to their maximum emission rate at activation. Note that the reduction factor *m*(*t*,*t*′)≤1 is a dimensionless quantity. It may be interpreted, in general, as a reduction in the number of bioluminescent cells (some cells stop light emission and the others maintain a constant emission rate) or in the emission of single cells (all cells sustain emission with a decaying rate), or as a combination of both effects. For definiteness, we stick to the former interpretation, so that the number of bioluminescent bacteria *a*(*t*) at time *t* is given by

3.5 $$a(t)=\int_{0}^{t}m(t,t^{\prime })\frac{dq(t^{\prime })}{dt^{\prime }}\;dt^{\prime }$$

where d*q*=d*q*(*t*′)/d*t*′⋅d*t*′ is the number of bacteria that were activated at time *t*′ (in the infinitesimal time interval between *t*′ and *t*′+d*t*′). Choosing a different interpretation for the memory function *m*(*t*,*t*′), however, would only change the meaning of the quantity *a*(*t*), without affecting *per se* equations (3.5) and (3.8) (we postpone a thorough discussion of this point until after equation (3.8)).

We further assume that the memory function *m*(*t*,*t*′) is a simple step-wise function (the Heaviside function) of the time difference *t*−*t*′ which drops to zero after a characteristic ‘quenching time’ *τ*

3.6 $$m(t,t^{\prime })=\Theta (\tau -(t-t^{\prime })),$$

where *Θ*(*x*)=1 for *x*>0 and *Θ*(*x*)=0 for *x*<0.

According to equation (3.6), bacterial cells, once activated, keep emitting light at the same rate only for a typical time *τ*, after which further bioluminescent emission is terminated. Consequently, in our model the ‘quenching time’ *τ* is assumed to be the same for all bacterial cells, possibly because of a tight metabolic control over the amount of reactive oxygen species in the bacterial ‘milieu’ [31,32], governed by an oxygen quenching mechanism. Note that this quenching mechanism prevents light emission from otherwise quorum-active cells.

According to equations (3.5) and (3.6), the number of bioluminescent bacteria at time *t* is then given by

3.7 $$a(t)=q(t)-q(t_{0})=f(t)n(t)-f(t_{0})n(t_{0}),$$

where $t_{0}=\max\{0,t-\tau \}$. As *q*(*t*) is an increasing function of time, *a*(*t*) can never be negative.

A key role is played by the luciferase enzyme which catalyses photon emission. There is biochemical evidence that the QS response removes the constitutive inhibition of luciferase expression that is mediated by negative feedback loops involving small regulatory RNAs [53].

Finally, we hypothesize that the number *p*(*t*) of photons emitted per second by the bioluminescent colony, which is eventually measured as the radiant flux in the climate chamber at nearly constant temperature and humidity [30,33], is simply proportional to that of bioluminescent bacteria *a*(*t*), as given by

3.8 $$p(t)=\kappa_{e}c_{e}a(t)=\kappa_{e}c_{e}[f(t)n(t)-f(t_{0})n(t_{0})].$$

The parameters involved are the enzyme copy number *c*_*e*_, the number of luciferase molecules expressed in a single bioluminescent bacterium and *κ*_*e*_, the luciferase turnover number in the photon emission reaction.

We recall that, as the memory function *m*(*t*,*t*′) is interpreted as a reduction in the number of bioluminescent cells, the emission rate of a single cell is assumed to be constant before quenching. Accordingly, (3.8) implies that the emission rate is the same for all cells, namely *k*_*e*_*c*_*e*_. The choice (3.6) for the memory function implicates then a quenching time *τ* homogeneous for all cells. A different choice, namely a continuous decay in time, would instead entail a heterogeneous quenching time.

On the other hand, a continuous decay could be associated with a homogeneous quenching behaviour, in the case that the memory function is interpreted as a reduction in the photon emission rate shared by all single bioluminescent cells. Equations (3.5) and (3.8) would still hold, with *k*_*e*_*c*_*e*_ defined as the maximum photon emission rate achieved just after activation, and *a*(*t*) defined as the effective number of bacteria that would produce the observed signal at time *t*, if emitting photons with such maximum rate.

In summary, our simplified model is then summarized by the five equations, namely (3.1), the Gompertz law of growth for the number of bacterial cells *n*(*t*); (3.2), the evolution equation governing the increase in AI concentration *c*(*t*); (3.3), which yields the fraction *f*(*t*) of quorum-active bacteria; (3.7), defining the number of bioluminescent bacteria *a*(*t*); (3.8), which represents the total rate of emitted photons *p*(*t*).

The meaning and the values reported in the literature, when available, for the parameters used in equations (3.1), (3.2), (3.3), (3.7) and (3.8), are summarized in table 1.

**Table 1.** Meaning and known values of the parameters used in equations (3.1), (3.2), (3.3), (3.7) and (3.8).

parameter	meaning	value
*α* (molecules cell^−1^ s^−1^)	AI production rate [51]	0.5 (AI-2) − 6.7 (AI-1)
*β* (h^−1^)	AI degradation rate [51]	0.0133 (AI-1) − 0.108 (AI-2)
c* (nM)	AI concentration threshold [51]	23 (AI-1) − 10-100 (AI-2)
*V* (μl)	volume of the colony spot [30]	10
*τ* (s)	bioluminescence ‘quenching’ time	—
*c*_*e*_ (molecules cell^−1^)	luciferase copy number [54]	7.82×10^4^
*κ*_*e*_ ($\frac{\mathrm{photons}}{s\;\;\mathrm{enzyme}}$)	luciferase turnover number [55]	0.04−0.6
*K* (dimensionless units)	carrying capacity	—
*μ* (h^−1^)	specific growth rate	—
λ (h)	lag time	—
*δ* (dimensionless units)	cooperativity of AI binding [56]	1 (non-cooperative)
*n*(0) (cells)	initial number of bacteria [30]	∼10^6^

### 3.3. Rescaled model equations

The experiments described in [30] measured the radiant flux *R*(*t*), that is the rate at which the electromagnetic radiation is emitted per unit time by bioluminescent bacterial colonies. The number of emitted photons per unit time *p*(*t*) predicted by model equations can then be used to fit the outputs of that set of experiments.

The fitting, if successful, does not allow to infer all the 11 model parameters *n*(0), *K*, λ, *μ*, *α*, *β*, c*, *δ*, *τ*, *c*_*e*_ and *κ*_*e*_. Indeed, by writing effective equations for dimensionless variables, it is easy to see that three out of the previous 11 parameters are redundant. By defining

3.9 $$\tilde{n}(t)=\frac{n(t)}{n(0)},\;\tilde{c}(t)=\frac{c(t)}{c^{*}},\;\text{and}\;\tilde{a}(t)=\frac{a(t)}{n(0)}$$

the original model equations become

3.10 $$\begin{array}{r} \log_{10}(\tilde{n}(t))=K\;\exp\;\left[-\exp\;\left(-\;\frac{\mu \cdot \exp(1)}{K}(t-\lambda )+1\right)\right] \end{array}$$

3.11 $$\begin{array}{r} \frac{d\tilde{c}(t)}{dt}=\tilde{\alpha }\tilde{n}(t)-\beta \tilde{c}(t) \end{array}$$

3.12 $$\begin{array}{r} f(t)=\frac{1}{1+{\left(1/\tilde{c}(t)\right)}^{\delta }} \end{array}$$

3.13 $$\begin{array}{r} \tilde{a}(t)=f(t)\tilde{n}(t)-f(t_{0})\tilde{n}(t_{0}) \end{array}$$

3.14 $$\begin{array}{r} \;\text{and}\;p(t)={\tilde{\kappa }}_{e}\tilde{a}(t)={\tilde{\kappa }}_{e}[f(t)\tilde{n}(t)-f(t_{0})\tilde{n}(t_{0})], \end{array}$$

where $t_{0}=\max\{0,t-\tau \}$. The effective rescaled parameters are

3.15 $$\tilde{\alpha }=\frac{\alpha n(0)}{Vc^{*}},\;{\tilde{\kappa }}_{e}=\kappa_{e}n(0)c_{e}.$$

The overall signal *p*(*t*), describing the activation and the subsequent quenching of bioluminescent emission, can be split into two contributions

3.16 p(t)=g(t)+h(t),

the ‘growth’ contribution, due to the increase in the number of bacterial cells in the colony

3.17 $$g(t)={\tilde{\kappa }}_{e}[\tilde{n}(t)-\tilde{n}(t_{0})]f(t),$$

and the ‘quorum activation’ contribution, due to the increase in the fraction of active cells

3.18 $$h(t)={\tilde{\kappa }}_{e}[f(t)-f(t_{0})]\tilde{n}(t_{0}).$$

### 3.4. Parameter fitting

Six different replicas of bioluminescence monitoring experiments were reported in [30]. Although the experimental procedure described in §2.2 was thoroughly replicated in the six experiments, it is well known that the resulting growth curves can, in general, display some biologic variability, due, for example, to the temperature history and the physiological state of initial cells [57]. As a result, the detected bioluminescent signals are also different for the six replicas (figure 1). In particular, the two parameters known to be mostly affected by biological variability are the lag time λ [57,58] and the carrying capacity (or growth yield) *K* [58]. The latter parameter is related to the asymptote $n(\infty )=n(0)\times 10^{K}$ reached by the number of bacteria at saturation for t→∞. The other growth parameters, the initial number of bacterial cells *n*(0) and the maximum specific growth rate *μ*, as well as the biochemical parameters *α*, *β*, c*, *δ*, *τ*, *c*_*e*_, *κ*_*e*_, are instead hypothesized to be the same in all the experiments.

Therefore, we adopted the strategy of fitting simultaneously the six curves of Delle Side *et al*. [30] by using 18 parameters: six parameters, *μ*, $\tilde{\alpha }$, *β*, *δ*, *τ*, ${\tilde{\kappa }}_{e}$, are common to all six experimental curves, whereas two quantities, *K* and λ, are allowed to change from one experiment to the other. The fitting was performed through a simulating annealing Monte Carlo procedure [59] which employed the Aarts cooling schedule [60]. The mean square deviation of the numerical solutions of equations (3.10)–(3.14) from the experimental points *R*_exp_(*t*), over all six curves was minimized in the 18*d* parameter space.

## 4. Results

The experimental points together with the best numerical fits are shown in figure 1, while the corresponding optimal parameters are listed in tables 2 and 3.

**Table 2.** Values of the biochemical parameters, common to all six experiments, obtained by fitting equations (3.10)–(3.14) to the experimental data.

parameter	value
${\tilde{\kappa }}_{e}$ (photons s^−1^)	2.42×10^10^
$\tilde{\alpha }$ (s^−1^)	3.95×10^−6^
*β* (h^−1^)	0.21
*μ* (h^−1^)	0.358
*δ* (dimensionless units)	1.07
*τ* (s)	32

**Table 3.** Values of the colony growth parameters that are assumed to be different in each experimental curve, as obtained by fitting equations (3.10)–(3.14) to the experimental data.

parameter	run1	run2	run3	run4	run5	run6
*K* (dimensionless units)	1.58	1.72	1.51	1.52	1.41	1.47
λ (h)	0.23	0.17	2.27	1.85	3.00	2.07

The quality of the fits as shown in figure 1 is quite good, with an average root mean square error (RMSE) between experimental data and theoretical fits of 2.478×10^7^ photons s^−1^. This result corroborates the validity of the modelling expressed by equations (3.10)–(3.14). As regards the parameters obtained with the fitting procedure, several aspects can be underlined. The fitting procedure works rather well despite the fact we use only 18 parameters instead of the 48 possible ones, if one considers 8 independent parameters for each experiment. This implies that our choice of keeping fixed six parameters for all the curves is consistent with the experimental findings.

### 4.1. Comparison with Gumbel fits

As can be seen in fig. 3 of Delle Side *et al*. [30], the experimental data can be nicely fitted using a Gumbel distribution function, which is known to be related to extreme-value statistics [61], and was conjectured to be related to the averaging of spatially correlated degrees of freedom [62]. On the other hand, the simple biophysical model presented here is able to reproduce experimental data convincingly, without involving either extreme values or any spatial dependence. As a matter of fact, the RMSE obtained by fitting a three-parameter non-normalized Gumbel distribution (mean, variance, height) to each experimental curve is 2.564×10^7^ photons s^−1^, being thus larger than the one (2.478×10^7^ photons s^−1^) obtained by fitting the parameters of the biophysical model given by equations (3.10)–(3.14). It should be noted that the two compared fits use the same overall number, 18, of parameters. However, the 6 different Gumbel fits are performed independently, each in a 3*d* parameter space, whereas the biophysical model fit is performed at once for the 6 different experimental curves in an 18*d* parameter space.

In fact, the similarity of the bioluminescence experimental curves to the Gumbel function could be related to the growth of bacteria colonies over time that generally follows a sigmoidal law. The Gompertz function, widely used in this context, represents exactly the cumulative distribution of Gumbel statistics.

### 4.2. Fit parameters are consistent with previously known values

The value of the degradation parameter *β* reported previously [50] is roughly twice as much as the one obtained from the biophysical model fit, in particular for the AI-2 signal molecule in *V. harveyi* [51]. However, one should consider the possibility that AIs are both chemically and enzymatically degraded and that AI chemical stability depends on pH [50]. In particular, a 0.3 increase in pH would result in the doubling of the degradation rate.

The value of the parameter *δ* is nearly 1, pointing to a *de facto* non-cooperative AI sensing upon receptor binding, as previously reported [56].

From the value of $\tilde{\alpha }$, it is possible to extract the rate of AI production per cell *α*. In fact, *n*(0)∼10^6^ as established with optical density measurements [30], c* is known [63] to be ranging from dozens to hundreds nM. Knowing the volume of the colony spot *V* =10 μl, we can conclude our estimate of *α* could range from 0.24 (if c*=10 nM) to 2.4 (if c*=100 nM) AI molecules produced per cell per second. This is somehow lower than the figure of 3–5 AI molecules produced per cell per second, generally reported for basal SI production [64,65], yet it would be consistent with what is known for the AI-2 signal molecule in *V. harveyi* [51].

The value of ${\tilde{\kappa }}_{e}$ obtained from the fit enables us to evaluate the luciferase copy number *c*_*e*_, that is the number of luciferase molecules present in a bioluminescent cell. In fact, $c_{e}={\tilde{\kappa }}_{e}/\kappa_{e}n(0)$, where the turnover number, *κ*_*e*_=0.04–0.6 photons emitted per second per luciferase molecule, may depend on the length of the aliphatic aldehyde fatty acid substrate [55] and *n*(0)=10^6^ is the initial number of bacterial cells already used above. The final estimate for the luciferase copy number is approximately 4.0 × 10^4^−6.1 × 10^5^, a fully reasonable estimate when compared with experimental data for bacterial luciferase [54]. Note that in the above comparison, one needs to know the relationship equation (3.15) between the rescaled parameters estimated from the fitting procedure and the unrescaled parameters known from the literature.

The estimate of the bioluminescence ‘quenching time’ *τ*=32 s implies that only a few reactions (at most 20, if *κ*_*e*_=0.6 photons emitted per second per luciferase molecule [55]) are catalysed by a single luciferase enzyme. Furthermore, this observation is consistent with what was already reported about the extremely slow turnover of bacterial luciferase [66].

Overall, we can conclude that our modelling of QS mechanism reproduces accurately the experimental results by using several parameters whose values are, in all cases, consistent with what is known from the literature.

### 4.3. Splitting the bioluminescent signal

**Figure 2.**
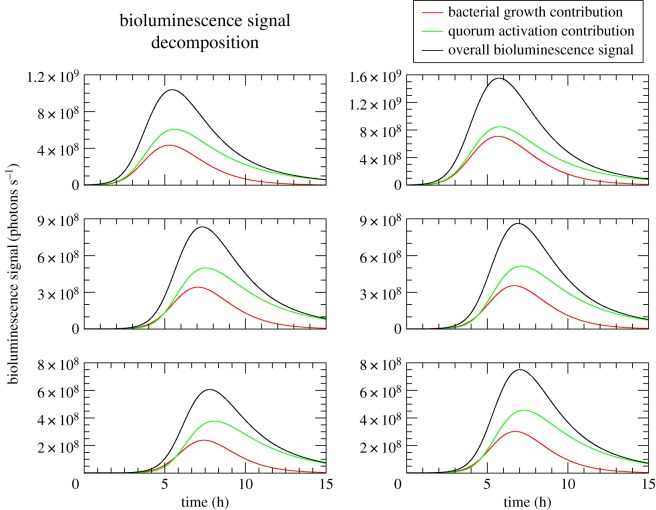
Decomposition of the overall bioluminescence signal *p*(*t*) (black) from biophysical model fits in colony growth *g*(*t*) (red) and quorum activation *h*(*t*) (green) contributions. Different panels show signal decomposition for the fits of the different experimental curves measured in [30].

Equations (3.16)–(3.18) can be used to check a further aspect of QS dynamics, namely the trade-off between colony growth and AI’s production. In figure 2 we plot the two contributions to the radiant flux, *g*(*t*) and *h*(*t*), the former is related to the growth of the bacterial colony, whereas the latter is related to the increase in the fraction of active cells, e.g. to the microscopic QS response mechanism. Figure 2 shows that these two mechanisms are not acting on different time scales. Instead, they contribute to the bioluminescence response at roughly the same time, with the fraction of active cells variation peaking slightly behind and slightly higher than the colony growth variation. In the long time tail of the radiant flux distribution, however, the quorum activation contribution is the dominant one. These features are consistently reproduced over all the six fits.

## 5. Discussion

It was previously observed that the same experimental data studied here can be fitted with a Gumbel function [30]. Yet, that was just an empirical fit and could not be explained by a predictive model such as the one we propose in this paper. Although the Gumbel distribution occurs in extreme value statistics, in bacterial QS no underlying extremal process is known to take place. On the other hand, it had been conjectured [62] that Gumbel statistics can originate in systems with spatially averaged critical properties. One could then speculate that bacterial colonies may represent an example of such a system [30]. Our present results show instead that a simple modelling of the QS mechanism is sufficient to reproduce the experimental data with a slightly higher accuracy, without relying on any unproven conjecture or unknown mechanism. The connection of the experimental curve with the Gumbel function can be rationalized by observing that the bacterial growth curve is modelled by a Gompertz function, e.g. the integral of the Gumbel function [33].

Having established a model that relies on a simple biochemical interpretation and quantitatively reproduces experimental data, we can then put forward some interesting observations.

First, in our simple dynamical model several features, well established for QS systems, were neglected, such as the positive feedback on AI production [6], the integration of signals from different AIs in the QS cascade [56], and the strongly heterogeneous bioluminescent response at single cell level [67]. The ability of our model to quantitatively and consistently reproduce the experimental signal is therefore remarkable. Although the above features should be considered in a more realistic and sophisticated model, they are not necessary to rationalize the time evolution of the bioluminescent signal produced by a growing bacterial colony, at least within the set-up of Delle Side *et al*. [30].

Second, it has to be stressed that the concerted behaviour observed in figure 2, with the growth of the bacterial colony and the increase in the fraction of active cells contributing with roughly equal amplitude and timing to the bioluminescent signal, is not the only *a priori* possible outcome of the experiments. Indeed, one could have, in principle, two alternative scenarios as limiting cases. In the first one, say at very high effective AI production rate $\tilde{\alpha }$, AI concentration would reach the threshold c* at a time *t*_1_ smaller than the lag time λ. In such a case, the overall signal would present a first smaller peak at *t*_1_ (because *n*(*t*) would still be small) followed by a main second one at around λ due to *g*(*t*), the contribution of *h*(*t*) being negligible (when the Hill function is already nearly constant and close to 1, for *t*≫*t*_1_).

Conversely, at low enough effective AI production rate $\tilde{\alpha }$, the first increase in the concentration of signal molecules is achieved because of the growth of the colony, prior to quorum activation. This peak would thus be small, because *h*(*t*)≪1. The main peak in the radiation flux should then occur at *t*_2_>λ, driven by *h*(*t*), the contribution of *g*(*t*) being negligible due to the saturation of the bacteria population *n*(*t*) for *t*>λ.

For even lower values of the effective AI production rate, no radiation flux would be eventually detected, when the saturation AI concentration *αn*(0)×10^*K*^/(*βV*) at the end of the colony growth is still below the quorum threshold c* and the colony never gets activated.

For the practical realizations of the above-described scenarios, one should consider that the distinction between active/inactive cells (for AI concentration above/below the quorum threshold) becomes less sharp in the case of essentially non-cooperative AI/receptor binding, as is ours.

Finally, we highlight that according to the model that we present here, different cells start bioluminescent emission, upon QS activation, at different times. Each cell emits photons with the same rate for approximately 30 s after its own activation and then ceases further emission.

The details of how we modelled the quenching mechanism, in particular the choice of a step-wise function equation (3.6) for the ‘memory function’, are somehow arbitrary, and were chosen to optimize the computational effort in the fitting procedure. An alternative choice, more computationally costly, could have been, for example, an exponentially decaying memory function after activation ($m(t,t^{\prime })=\exp[-(t-t^{\prime })/\tau ]$).

However, the very existence of a quenching mechanism with a short characteristic time is crucial to obtain a reliable fit to the experimental data. Note that the quenching time is much smaller than the other time scales in the model. Therefore, we argue that any memory function with a unique short characteristic time would fit the experimental data equally well.

On the other hand, the interpretation of the memory function discussed in §3.2 cannot be decided based solely on our fitting. Accordingly, the quenching behaviour could either be the same or vary across different cells in the colony. Equation (3.6) implies the former possibility, whereas the latter would correspond to an exponentially decaying memory function that describes a reduction in the number of bioluminescent cells. To discriminate between the two scenarios remains an interesting open question.

From a biological perspective, the very small value of the ‘quenching time’ *τ* makes a quenching mechanism based on luciferase turnover unlikely. On the other hand, it is intriguing to hypothesize that the bioluminescence quenching that we observe here could be due to a switch in the type of luciferase activity, driven by the high amount of reactive oxygen species in the highly drying environment where the experiments were carried out [30]. As a matter of fact, it had been previously reported that *V. harveyi* luciferase, but not necessarily the process of light emission, may be involved in the detoxification of reactive oxygen species, thus playing a role in the protection of cells against oxidative stress [32]. This role had also been suggested to be at the origin of the development of marine bioluminescence [31].

## 6. Conclusion

We proposed a model for the QS mechanism that closely reproduces experimental data on bioluminescence by *V. jasicida*, within the set-up of Delle Side *et al*. [30]. By using a simulated annealing procedure, we obtained biochemical and growth parameters that are in good agreement with the literature data. The results of our numerical fit clearly suggest that the activation of the quorum mechanism is intertwined with the rapid growth of the bacterial population. In other words, the bioluminescence response acts exactly as a proxy to sense the rapid growth of the colony. This concerted trade-off could be simply due to the choice of the initial bacterial cell density in the experiments, or rather enforced by a feedback mechanism that tunes the sensitivity of the QS response to AI concentration, as already reported for *V. harveyi* [53]. Understanding whether the latter mechanism operates also in the experimental set-up considered in this paper is a subject that deserves further investigation. Moreover, a distinctive novel feature of the proposed model is the presence of a short (approx. 30 s) bioluminescence ‘quenching’ time, crucially needed to explain the observed data. Although the quenching time in our numerical fits is assumed to be the same for all activated cells in the bacterial colony, heterogeneous quenching behaviour could as well be introduced within the framework proposed here. The nature of single cell quenching behaviour and its hypothesized connection with the protective role of bacterial luciferase against reactive oxygen species are issues that call for further experiments.

## Data accessibility

All relevant data are within the paper.
